# Hygienic Treatment of Patients

**Published:** 1868-03-15

**Authors:** J. Little

**Affiliations:** Le Roy, Ill.


### CHICAGO MEDICAL JOURNAL.

*Vol. XXV.—MARCH* 15, 1868.—*No.* 6.

### HYGIENIC TREATMENT OF PATIENTS.

BY J. LITTLE, M.D., LE ROY, ILL.

(*Concluded from p.* 176.)

DURING the discussion of typhus fever before the Academy of Medicine in New York city, a few years ago, Dr. Smith related the following brief history. During the year 1838, a vessel came into port, some where south of the city, with typhus fever raging among the passengers and crew to a fearful extent. The passengers and vessel were in a shockingly filthy condition, as the voyage had been a protracted one, and many deaths had occurred, though the sick received prompt medical aid. As soon as the vessel landed, the sick were immediately taken on shore, except four of the crew, who preferred to remain on the ship, as they had comfortable quarters and good attention. Tents and shelters were put up for the sick, and pure air, and good water and food were abundantly supplied. All were bathed daily, and clean, comfortable clothing and bedding were furnished as often as occasion required. Sweet milk, fresh meats and animal essences, and fruits and vegetables were liberally provided and administered to all. No medicines at all were given, except a very mild cathartic, or an opiate, to a very few, and a little wine to the most feeble. The first night the patients were ashore, and before shelters and beds were provided, a heavy shower of rain fell, drenching every one completely, but the succeeding day being warm and sunny, every thing was soon dried, and the patients made very comfortable. The number treated on land was about sixty, if I remember correctly; and of these but three or four died, and of the four treated on the vessel three died. The sixty were treated hygienically, the four therapeutically; the sixty were removed from the cause of the disease, and placed in harmony with nature's agents and laws, the four were left under the full power of the cause of the disease, and dosed instead of nourished, and we have the result.

In the years 1863 and 1864, typhus fever prevailed in New York city, and the fever wards of Bellevue Hospital were filled with typhus patients. Sixteen of the house staff had the disease, and eight died; and sixteen of the attendants had fever, and nine died. These cases were all treated on the most approved and scientific plans, by the ablest and most experienced physicians of the city of New York. Stimulants, the mineral acids, quinine, etc., were freely used.

Prof. Hamilton had just returned from the army, and he conceived the idea of removing all the typhus fever patients to Blackwell's Island, and placing them in new tents and treating them on purely hygienic principles. This was accordingly done, and at the end of six months, a report was made by rof. Loomis, the attending physician, which showed a mortality of one in ten. No medicines at all had been used as *remedies* for the disease. Abundance of pure air was supplied day and night; all clothing, furniture, and vessels were kept scrupulously clean; the patients were bathed and well nourished, daily. Cheerfulness was encouraged, and every thing was managed on strictly hygienic principles.

This was a brilliant triumph of hygiene, and Prof. Loomis' report was commented upon largely, both in this country and Europe. This report was a history of an occurrence, and nobody could criticize or gainsay it.

I witnessed several striking instances in the army, during the late war, in which treating patients hygienically was far superior to treating their diseases with drugs and medicines merely. In the autumn of 1861, typhoid fever raged extensively, and proved very fatal among the troops at Ironton, and Pilot Knob, Mo. The sick were treated in hospital tents, an old school-house, a church, and two or three old store-houses. These places were all crowded full of the sick, were filthy, and poorly ventilated. The disease was vigorously treated, according to Wood and Watson's practices, but no special attention was paid to sanitary regulations. *Opium*, *Blue pill*, *Ipecac*, *Turpentine emulsion*, *Quinine*, *Sinapisms*, and other medical agents were daily exhibited, and still death swept its victims to premature graves. The number of deaths was so large as to attract the attention of the Sanitary Commission at St. Louis, and agents were sent to investigate the matter. In their report the agents attributed the large mortality to non-attention to sanitary rules in the treatment of the disease and the management of the hospitals. In compliance with their recommendation, a complete and radical change was at once made in the treatment of the sick. The hospitals were all thoroughly ventilated and cleansed, the patients were provided with clean, comfortable beds and clothes, a sufficiency of fresh milk, eggs, beef, bread, fruits, and vegetables were furnished, warm sponge baths were daily given, and an air of cheerfulness was made to take the place of the gloom and sorrow that filled every heart, and every thing was managed in a hygienic and common sense way. Medicines were very sparingly administered, and the death rate decreased from one in six to about one in fifteen. Common sense would have dictated this change long before, if the doctors had not been blinded by the theories and routinism of the books.

Every camp and hospital in the army afforded proof of the safety and advantage of treating patients hygienically, as well as their diseases therapeutically. In the history of the small-pox hospital of Vicksburg, we have a marked example of the superior efficacy of the hygienic management of the patient, over mere drug medication, in variola.

In the practice of many of our brethren, we see the disease treated most persistently and vigorously, to the almost total neglect of the laws of health and life. The patient is allowed to lie in the same bed, clothes, and room, from week to week; the doctor, in the mean time, making his daily visits, and prescribing pills, powers, drops, decoctions, etc., and giving strict orders how they shall be given, and not a word is said about ventilation, cleanliness, and dietetics. Treat a strong, healthy man as the sick are too often treated; confine him in a close room, in a warm bed, and deprive him of suitable food, and drink, and agreeable company and surroundings, and how long will he continue well? When physicians learn that all the natural agents essential to health and life, are to be supplied in their freshness and purity to the sick; when they learn what is good for a well man is not injurious to a sick man, they will treat their patients more, and their diseases less, heroically.

Why cram medicines down our patients' throats on every occasion? Is it treating our fellow citizens fairly to dose them every time they ask our advice? The physician who prescribes medicines when nothing is needed, in order to mislead his patient and make money, is too base to perform the sacred offices of the noble profession he is prostituting. Hundreds of patients have been destroyed by the drugs given them to cure their diseases. Is it not, indeed, astonishing what quantities of drugs and medicines are annually consumed by the invalids of our country? Patent medicines of almost innumerable kinds fill the shelves of the drug-stores in the land, and every newspaper has from one to four or five columns of advertisements and recommendations of these villainous compounds. Why is this? Is it not because the people have a ridiculous and superstitious faith in medicines, growing out of their ignorance of the science of medicine? Are not the physicians more responsible for all this than any other class of community? Who have kept the people in ignorance of the science of medicine, and taught them to have so much faith in drugs and medicines, if they have not? If the people knew as much of anatomy and physiology as the doctors, do you suppose they would swallow every nostrum that is blown at them by quacks? They would not take medicines at all, unless prescribed by a regularly educated physician. Let physicians deem it not beneath their dignity to give the people all the information possible on medical topics, and they will have less faith in medicines, but a great deal more in the scientific physician.

Prof. Meigs says "a physician is not of necessity a doser, a drugger; and that in a large number of cases in which he is consulted, the patient will escape all physic and be cured by wise counsel. It is often dangerous to ask a physician the question what shall we do; because habit, custom, routinism almost always compel him to say take—take. Every one knows very well that a large variety of the complaints of pains, disabilities and fevers made by physicians, require for their removal only that the patient should know their nature, causes, and tendencies. The homœopaths treat multitudes of people by thus giving them not the least particle, but only the name of a drug, and all that recover under their guidance, give evidence of the great abundance of spontaneous cures."

I believe many are driven to homœopathy because they are tired of being drugged with massive doses of powerful medicines every time they are ill, or even "slightly indisposed." If there is such necessity for medicines, and they possess such wonderful virtues, why is it that the homœopaths, hydropaths, electricians, and others who discard them entirely, herald so many cures, and have so many followers?

If we would study nature as did the fathers of medicine, observe the natural course of diseases uninfluenced by drugs, we would soon learn that almost all diseases tend to recovery. Any experienced physician will acknowledge, if he is honest, that not more than one patient in five who consult him really needs any thing more than hygienic treatment. It is now admitted on all sides that the practice of our art is not nearly so heroic as it was twenty years ago, and it is also admitted to be more successful than then. The tendency is to conservatism, and to dispense with these powerful measures so freely employed in years gone by.

Prof. Flint says: "It may be laid down as a golden rule in therapeutics, that active measures of treatment are only to be employed in cases to which they seem to the physician to be clearly indicated. The severity of the disease, and the danger of the patient, be they never so great, do not alone constitute grounds for the employment of active measures. If they are not useful they will be likely to do harm. The physician should be content with doing nothing when ignorant how to do good." These are truly golden rules, and there are many more such in Prof. Flint's new work on the theory and practice of medicine. In the treatment of all diseases he dwells more or less at length on their hygienic management, and in some cases he says, that "far more reliance is to be placed on hygienic than on medical measures of treatment." I have heard him declare, on more than one occasion, his preference for hygieo-therapeutics, if compelled to choose between that and therapeutics strictly so called.

Prof. Bedford, one of the most distinguished and successful practitioners and teachers of New York, always warmly urged the immense value of hygiene in the treatment and prevention of all classes of disease. All who have studied the writings of that illustrious physician of Scotland, Prof. Bennett, know what great stress he puts on sanitary and hygienic measures. I believe the leading medical men every where have more confidence in purely and strictly hygienic and sanitary agents and measures, than they have in drugs and medicines alone in the cure and prevention of diseases.

I heard a very learned and experienced Irish physician once remark, that he used but little medicine in his practice of late, and when he did use it, it was a good deal as cities use scavengers — to clean the streets, alleys, and channels of his patient's system. He said people were continually violating nature's laws, and bringing down upon them their penalties, and it was necessary to remove the obstructions to normal actions in the body, and this could sometimes be most speedily done with appropriate medicines, but the cure of the patient was the work of nature, and the physician who did not avail himself of hygienic measures in the treatment of his patients was not wise, for in this field were his grandest and safest resources.

We learn from our journals, that some of the most scientific physicians of Germany and England are treating their patients with milk. This milk treatment seems to be gaining a good deal of popularity on account of its success and agreeableness. I read an article in the *London Lancet*, a short time since, in course of which the writer used the following language: "Milk, in the treatment of all fevers, is a valuable agent, especially in typhus and typhoid fevers. Drugs complicate and mask the disease. Leave as many cases of fevers as possible to their natural course, unaffected by drugs or stimulants. Patients with fevers must be fed, and fed often, and with milk. Sweet milk or butter milk may be employed. Withhold brandy and wine, and all alcoholic liquors, and give milk."

Dr. Parish, of New York, in an address before the Academy of Medicine, said he believed that many typhus fever patients had died drunk, and others of delirium tremens, so freely had wine, brandy, etc., been administered in the treatment of this disease. He said that over-medication was so common that it was next to impossible to study the symptoms of disease, and their natural history. Dr. Hooker believes that the typhus syncopalis of a preceding generation in New England, "was often, in fact, a brandy and opium disease." It is said that England's greatest surgeon, Sir Astley Cooper, once remarked that more harm than good is done by medicines, and other surgeons and physicians of renown have made similar remarks.

Everybody knows, who has ever taken a dose of powerful medicine, or has seen a person under its influence, that it is directly hurtful, though it may be indirectly beneficial.

A very able and facetious professor once declared that "medication without insuring favorable hygienic conditions, is like amputation without ligatures." He said, "throw out opium, throw out a few specifics, throw out wine which is food, and the vapors which produce anæsthesia, and I firmly believe that if the whole Materia Medica, *as now used*, could be sunk to the bottom of the sea, it would be all the better for mankind—and all the worse for the fishes." Further on in the same address, he says, "but if the Materia Medica were lost overboard, how much more pains would be taken in ordering all the circumstances surrounding the patient, than are taken now by too many who do their duty and earn their money when they write a prescription for a patient left in an atmosphere of domestic malaria, or to the most negligent kind of nursing. I confess that I should think my chances of recovery from illness less with Hippocrates for my physician, and Mrs. Gamp for my nurse, than if I were in the hands of Hahnemann himself, with Florence Nightingale or good Rebecca Taylor to care for me."

Time and faith in God are the great panaceas for all the troubles and evils of this life. "What can not be cured must be endured," and there are diseases or cases that hygienic and medical treatment will fail to remove, and the patient will have to suffer on till death comes to his relief.

To study nature, to be in harmony and sympathy with her, to imitate her in all we do, to question her at every step, should be the aim and glory of the physician. The art of medicine is but the hand-maid of nature, and the true physician her humble, dutiful, intelligent and *free* servant. Then, gentlemen, with hygiene as our professional compass, and "*experientia et progressus*" as our motto, and having a living faith in the principles of Christianity, our paths through life will be thickly strewn with living witnesses of our usefulness and success as physicians, and our goodness as men.

